# CT-based nomogram for predicting EGFR mutation status in ground-glass nodules of lung adenocarcinoma

**DOI:** 10.3389/fimmu.2025.1630119

**Published:** 2025-11-20

**Authors:** Li Li, Yantao Yang, Xinjie Zhou, Chen Zhou, Qiubo Huang, Jie Zhao, Yaowu Duan, Wangcai Li, Hong Yao, Liuyang Yang, Lianhua Ye

**Affiliations:** 1 Cancer Biotherapy Center & Cancer Research Institute, The Third Affiliated Hospital of Kunming Medical University, Yunnan Cancer Hospital, Peking University Cancer Hospital Yunnan, Kunming, China; 2 Department of Thoracic and Cardiovascular Surgery, The Third Affiliated Hospital of Kunming Medical University, Yunnan Cancer Hospital, Peking University Cancer Hospital Yunnan, Kunming, China; 3 The Third Affiliated Hospital of Kunming Medical University, Yunnan Cancer Hospital, Peking University Cancer Hospital Yunnan, Kunming, China

**Keywords:** ground glass nodule, radiologic characteristic, lung adenocarcinoma, EGFR, prediction model, nomogram

## Abstract

**Purpose:**

This study aimed to establish a nomogram based on computed tomography (CT) imaging characteristics to predict epidermal growth factor receptor (EGFR) mutation status in patients with ground-glass nodules (GGNs), thereby aiding medication decision-making.

**Materials and methods:**

In total, 935 patients diagnosed with GGNs were enrolled. Patients undergoing surgery from August 2019 to December 2023 (n=709) comprised the training cohort, whereas those treated between January 2024 and March 2025 (n=226) constituted the validation cohort. Clinical parameters and radiological features were recorded for all participants. The training group underwent univariate and multivariate logistic regression analyses to identify significant predictive variables, subsequently facilitating the construction of a nomogram prediction model. The model’s discrimination, calibration, and clinical applicability were validated in both patient cohorts.

**Results:**

Multivariate logistic regression analysis revealed maximum nodule diameter, consolidation-to-tumor ratio (CTR), mean CT values, presence of air bronchogram signs, and vascular convergence signs as independent predictors of EGFR mutations. The resulting nomogram demonstrated robust predictive capability, achieving an area under the curve (AUC) of 0.87 (95% CI: 0.85–0.90) in the training group and 0.87 (95% CI: 0.82–0.92) in the validation group. Bootstrap internal validation yielded an AUC of 0.89, confirming strong model discrimination. Calibration plots and decision curve analysis further supported the model had a good calibration degree and clinical practicability across both groups.

**Conclusion:**

The nomogram integrating maximum diameter, CTR, mean CT value, air bronchogram signs, and vascular convergence signs effectively predicts EGFR mutation status in GGNs, offering a valuable tool for clinical guidance and patient management strategies.

## Introduction

Lung adenocarcinoma remains among the malignancies with the highest global morbidity and mortality rates (1), and adenocarcinoma is recognized as its predominant histological form.

With advances in early screening for lung cancer, an increasing number of multiple primary lung adenocarcinomas (MPLC) presenting as GGNs have been identified (2). Surgical intervention remains the primary treatment method for these patients (3). However, after the primary lesion is resected, several management options exist for the remaining lesions (4, 5).

Targeted therapy is one of these treatment strategies (6). Prior research indicates that Epidermal Growth Factor Receptor (EGFR) mutations frequently occur in MPLC (7, 8). Cheng et al. (6) reported favorable clinical responses to EGFR-tyrosine kinase inhibitors (EGFR-TKIs) in residual pulmonary lesions. However, therapeutic outcomes vary due to genetic heterogeneity among lesions. Determining the EGFR mutation status of ground-glass lesions in advance has thus become essential for guiding medication decisions (6).

Currently, tissue biopsy-based genetic testing is regarded as the gold standard for detecting EGFR mutations in lung cancer patients (9). However, several limitations hinder its widespread use, including economic constraints, limited availability of advanced testing technology, small biopsy samples from minimally invasive procedures, poor physical condition of patients, suboptimal lesion locations, and the relatively low sensitivity of DNA sequencing instruments (10–12). Consequently, not all patients with primary lung cancer can successfully undergo genetic mutation testing. Moreover, the feasibility of performing genetic testing on every lesion in patients with MPLC presenting as GGNs is considerably low. Therefore, a non-invasive, efficient, and rapid method for evaluating the EGFR mutation status of ground-glass lesions is urgently needed.

Several studies have explored correlations between EGFR mutations in lung adenocarcinoma and specific clinical or imaging features. Zou et al. (13) identified ground-glass opacity (GGO) as an independent factor associated with EGFR mutations. Similarly, Hong et al. (14) observed that tumors harboring EGFR mutations exhibited a higher proportion of GGO features. Rizzo et al. (15), in an investigation involving 286 patients, observed air bronchograms in approximately 60% of EGFR-positive cases, significantly greater than the 35% observed in EGFR-negative tumors. Lee et al. (16) identified a significant association between air bronchogram and exon 21 missense mutations. However, Glynn et al. (17) observed no significant difference in the presence of air bronchogram between EGFR mutation-positive and mutation-negative groups. Following improvements in their methodology, Dai et al. (18) indicated that air bronchogram occurred more frequently in EGFR mutation-positive cases.

Some researchers have explored the relationship between EGFR mutations and tumor size. Rizzo et al. (15) proposed that smaller tumor diameter was significantly associated with EGFR mutation positivity. This finding aligned with the results of Hsu et al. (19) from a study of 149 patients. Conversely, Dai et al. (18) found no correlation between tumor diameter and EGFR mutation status. Paez et al. (20) indicated that EGFR mutation positivity was higher among non-smokers, females, and individuals of Asian descent, which was consistent with findings from other studies (21, 22). Moreover, certain studies have proposed a link between serum carcinoembryonic antigen (CEA) levels and EGFR mutations, suggesting that higher CEA concentrations correlate with increased mutation prevalence (23). However, Zou et al. (13) did not observe a significant correlation between CEA elevation and the presence of EGFR mutations.

Nevertheless, most existing studies primarily investigated the imaging features of advanced-stage lung adenocarcinoma, predominantly including solid lesions, which limits their applicability to GGNs. Although Ping et al. previously investigated GGNs, their study was limited by small sample size and insufficiently detailed analyses regarding clinical and imaging predictors. Thus, the present study utilizes a larger patient cohort to comprehensively evaluate clinical and radiological features that independently predict EGFR mutation status, aiming to inform clinical medication decisions and improve therapeutic outcomes.

## Materials and methods

### Participants

The institutional ethics committee approved this retrospective study (Ethics review number: KYLX2025-278) and waived informed consent requirements. Clinical records and chest CT images of patients undergoing surgical resection for GGNs at Yunnan Cancer Hospital from August 2019 to March 2025 were retrospectively reviewed.

Inclusion criteria comprised: (1) Availability of preoperative CT scans obtained within two weeks before surgery at the Third Affiliated Hospital of Kunming Medical University, identifying at least one GGN; (2) Surgical resection with histopathological confirmation of adenocarcinoma subtypes, including adenocarcinoma *in situ* (AIS), minimally invasive adenocarcinoma (MIA), and invasive adenocarcinoma (IAC), without evidence of lymph node metastasis or distant spread, accompanied by EGFR mutation status analysis; (3) No prior radiotherapy, chemotherapy, or other antitumor therapies for pulmonary GGNs; (4) Patients aged 18 years or older.

Exclusion criteria included: (1) Incomplete medical records or imaging data; (2) Pulmonary infections compromising image interpretation; (3) Severe respiratory motion artifacts affecting CT assessment; (4) Inconsistency between postoperative pathological findings and preoperative CT localization of GGNs.

**Figure 1:**
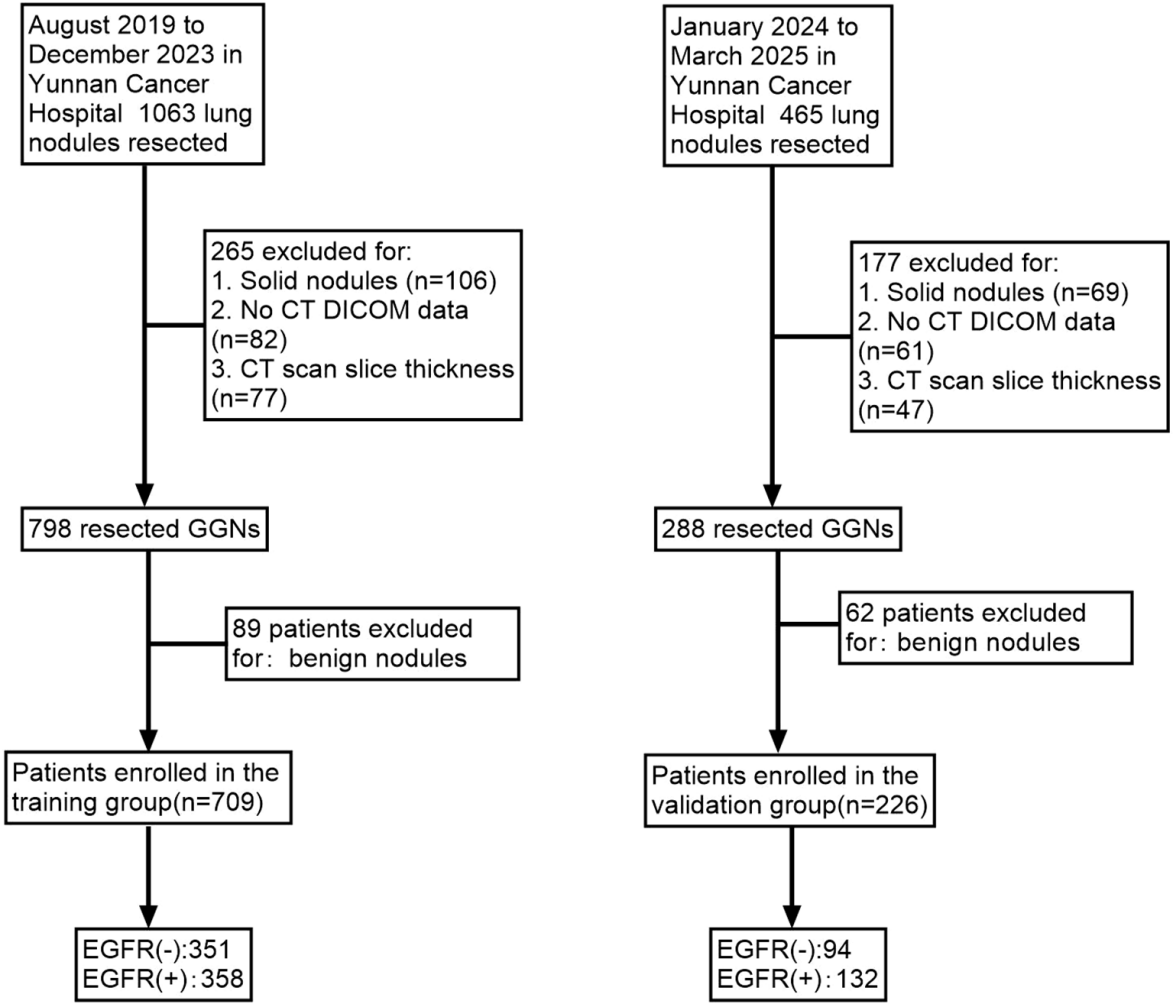
Patient screening flowchart.

Patients were classified into two cohorts based on surgical dates: a training group (709 GGNs resected from August 2019 to December 2023) and a validation group (226 GGNs resected from January 2024 to March 2025) (**Figure 1**).

### CT acquisition

Patients underwent breathing instruction before imaging. During CT scanning, each patient lay supine with arms raised overhead, holding breath at deep inspiration or quiet breathing. Spiral CT scans covering from lung apex to base were obtained using a Siemens 64-row, 128-slice CT scanner with parameters: tube voltage 120 kV, current 100 mAs, pitch 1.0, slice thickness 1 mm, and image matrix size 512×512. Images were reconstructed using a high-resolution lung algorithm (window width 1200–1500 HU, window level −600 to −700 HU) and standard soft-tissue algorithm (mediastinal window: width 400–500 HU, level 40–50 HU). All imaging parameters were derived from non-contrast CT scans.

### Image analysis

Two chest radiologists with over 15 years of diagnostic experience independently assessed all CT scans without prior knowledge of clinical or EGFR mutation data. Any discrepancies were resolved by consensus discussion. The following high-resolution CT (HRCT) imaging characteristics, both continuous and categorical, were evaluated on a Picture Archiving and Communication System:(1) Spiculation sign: nodular margin irregularities characterized by spike-like protrusions extending into surrounding lung parenchyma;(2) Lobulation sign: nodules exhibiting irregular margins with scalloped or undulated contours;(3) Vacuole sign: presence of air-density cavities measuring less than 5 mm within nodules, delineated by smooth boundaries;(4) Air bronchogram sign: visualization of air-filled bronchial structures traversing nodules continuously across multiple axial slices;(5) Vascular convergence sign: vessels near the nodules appearing convergent, attracted to, or concentrated around lesions;(6) Pleural traction sign: linear or star-shaped fibrous connections extending from the nodule to the pleura;(7) CTR: ratio calculated by dividing the largest solid-component diameter (lung window) by the largest nodule diameter;(8) Maximum diameter: greatest lesion diameter measured on axial CT slices (24); (9) Mean CT value: mean CT value recorded using a region-of-interest (ROI) cursor placed at the maximum cross-sectional area, avoiding prominent bronchial structures, blood vessels, or vacuoles.

### EGFR mutation detection

EGFR mutation testing (exons 18–21) was performed on lung adenocarcinoma tissues obtained from surgery. The testing methods included next-generation sequencing (NGS) and PCR-based amplification assays. Patients were categorized according to test results as either EGFR mutation-positive (+) or EGFR wild-type (-).

### Imaging feature selection

In the training group, clinical and radiological variables between EGFR(+) and EGFR(-) groups were compared using univariate analysis. Variables achieving statistical significance (P<0.05) underwent subsequent multivariate logistic regression to identify independent predictors of EGFR(+). Variance inflation factors (VIF) were calculated to assess multicollinearity among selected variables. Comparisons of clinical and imaging characteristics between the training and validation groups were performed using independent-sample t-tests, Mann–Whitney U tests and chi-square analyses.

### Model construction and performance assessment

Multivariate logistic regression was employed to evaluate combined predictive effects. Predictors demonstrating statistical significance in multivariate analyses (P<0.05) were integrated into a nomogram model. The predictive nomogram for EGFR mutations in GGNs was constructed using R software. Model discrimination was assessed through area under the receiver operating characteristic curve (AUC), calibration curves determined calibration accuracy, and decision curve analysis evaluated clinical utility in both cohorts. Internal validation involved 1,000 bootstrap resamples.

### Statistical methods

Continuous data with normal distribution were compared using independent-sample t-tests, while non-normally distributed continuous data were analyzed by Mann–Whitney U tests. Categorical variables underwent chi-square testing. Variables identified by univariate analyses (P<0.05) were entered into binary logistic regression, employing backward elimination to derive a final logistic regression model. SPSS (version 26.0) and R (version 4.4.1) statistical software were utilized. To avoid multicollinearity, a bidirectional stepwise regression was applied. Multicollinearity was assessed by calculating the variance inflation factor (VIF) for each predictor in the logistic regression model. A VIF threshold of 5 was considered acceptable, as values above this threshold indicate high multicollinearity. Optimal cutoff values for continuous predictors were determined by Youden’s index derived from the receiver operating characteristic (ROC) curve analysis. Decision curve analysis was used to assess the clinical utility of the predictive model at various threshold probabilities. For each threshold, net benefit was calculated by subtracting the proportion of false positives from the proportion of true positives, considering the relative harm of false positives and false negatives. The model’s performance was compared with two baseline strategies: “treat-all” and “treat-none.” Net benefit at each threshold was plotted, visualizing the decision curve to assess the model’s effectiveness in clinical decision-making. Statistical significance was defined as P<0.05.

## Results

### Clinical and imaging features

Overall, 935 patients (299 males [31.98%], 636 females [68.02%]) were included. No statistically significant differences emerged in clinical and imaging variables between training and validation cohorts, affirming their suitability for subsequent model development and validation analyses (**Table 1**).

**Table 1:** Comparison of clinical and CT features between the training group and the validation group.

Variables	Total (N = 935)	Training group (N = 709)	Validation group (N = 226)	*P*
Age (years)	51.95 ± 10.87	52.32 ± 10.96	50.76 ± 10.51	0.06
Mean CT value (HU)	-410.00 (-510.00, -300.00)	-420.00 (-500.00, -320.00)	-370.00 (-520.00, -250.00)	0.176
Maximum diameter (mm)	16.00 (13.00, 19.00)	16.00 (13.00, 19.00)	17.00 (12.00, 19.50)	0.676
CTR (%)	42.00 (25.00, 65.00)	41.00 (25.00, 65.00)	51.50 (20.75, 64.00)	0.798
Gender, n (%)				0.304
male	299 (31.98)	233 (32.86)	66 (29.20)	
female	636 (68.02)	476 (67.14)	160 (70.80)	
Smoking history, n (%)				0.533
No	680 (72.73)	512 (72.21)	168 (74.34)	
Yes	255 (27.27)	197 (27.79)	58 (25.66)	
CEA, n (%)				
Negative	820 (87.70)	622 (87.73)	198 (87.61)	0.962
Positive	115 (12.30)	87 (12.27)	28 (12.39)	
CA125, n (%)				
Negative	870 (93.05)	663 (93.51)	207 (91.59)	0.323
Positive	65 (6.95)	46 (6.49)	19 (8.41)	
Vacuole sign, n (%)				0.723
No	797 (85.24)	606 (85.47)	191 (84.51)	
Yes	138 (14.76)	103 (14.53)	35 (15.49)	
Spiculation, n (%)				0.957
No	531 (56.79)	403 (56.84)	128 (56.64)	
Yes	404 (43.21)	306 (43.16)	98 (43.36)	
lobulation, n (%)				0.889
No	653 (69.84)	496 (69.96)	157 (69.47)	
Yes	282 (30.16)	213 (30.04)	69 (30.53)	
Pleural traction sign, n (%)				0.84
No	708 (75.72)	538 (75.88)	170 (75.22)	
Yes	227 (24.28)	171 (24.12)	56 (24.78)	
Vascular convergence sign, n (%)				0.695
No	573 (61.28)	437 (61.64)	136 (60.18)	
Yes	362 (38.72)	272 (38.36)	90 (39.82)	
Air bronchogram sign, n (%)				0.47
No	797 (85.24)	601 (84.77)	196 (86.73)	
Yes	138 (14.76)	108 (15.23)	30 (13.27)	

### Analysis and selection of clinical and imaging features

Univariate analyses within the training cohort identified several imaging features, such as maximum diameter (P<0.001), CTR (P<0.001), and mean CT value (P<0.001), as significantly higher in the EGFR(+) group (**Table 2**). Additionally, imaging findings including lobulation, air bronchogram sign, vascular convergence sign, and pleural traction sign were more common in EGFR(+) group (P<0.05). Non-smokers also exhibited a higher frequency of EGFR mutations (**Table 2**).

**Table 2:** Relationship between clinical and imaging and EGFR mutation status of ground glass nodules.

Variables	Total (n = 709)	EGFR (−) (n = 351)	EGFR (+) (n = 358)	*P*
Age(years)	52.32 ± 10.96	53.04 ± 11.32	51.63 ± 10.57	0.086
Mean CT value (HU)	-420.00 (-500.00, -320.00)	-490.00 (-520.00, -430.00)	-340.00 (-410.00, -276.25)	<.001
Maximum diameter (mm)	16.00 (13.00, 19.00)	13.20 (12.00, 16.00)	18.00 (16.00, 21.00)	<.001
CTR (%)	41.00 (25.00, 65.00)	31.00 (22.00, 46.00)	57.00 (34.00, 67.00)	<.001
Gender, n (%)				0.089
male	233 (32.86)	126 (35.90)	107 (29.89)	
female	476 (67.14)	225 (64.10)	251 (70.11)	
Smoking history, n (%)				0.036
No	512 (72.21)	241 (68.66)	271 (75.70)	
Yes	197 (27.79)	110 (31.34)	87 (24.30)	
CEA, n (%)				
Negative	622 (87.73)	311 (88.60)	311 (86.87)	0.482
Positive	87 (12.27)	40 (11.40)	47 (13.13)	
CA125, n (%)				0.702
Negative	663 (93.51)	329 (93.73)	333 (93.02)	
Positive	46 (6.49)	22 (6.27)	25 (6.98)	
Vacuole sign, n (%)				0.524
No	606 (85.47)	303 (86.32)	303 (84.64)	
Yes	103 (14.53)	48 (13.68)	55 (15.36)	
Spiculation, n (%)				0.058
No	403 (56.84)	212 (60.40)	191 (53.35)	
Yes	306 (43.16)	139 (39.60)	167 (46.65)	
lobulation, n (%)				0.041
No	496 (69.96)	258 (73.50)	238 (66.48)	
Yes	213 (30.04)	93 (26.50)	120 (33.52)	
Pleural traction sign, n (%)				0.016
No	538 (75.88)	280 (79.77)	258 (72.07)	
Yes	171 (24.12)	71 (20.23)	100 (27.93)	
Vascular convergence sign, n (%)				<.001
No	437 (61.64)	238 (67.81)	199 (55.59)	
Yes	272 (38.36)	113 (32.19)	159 (44.41)	
Air bronchogram sign, n (%)				<.001
No	601 (84.77)	316 (90.03)	285 (79.61)	
Yes	108 (15.23)	35 (9.97)	73 (20.39)	

Multivariate logistic regression further confirmed maximum diameter (OR = 1.178, 95% CI:1.113–1.247), CTR (OR = 1.025, 95% CI: 1.015–1.035), mean CT value (OR = 1.010, 95% CI: 1.008–1.012), vascular convergence sign (OR = 1.632, 95% CI: 1.093–2.438), and air bronchogram sign (OR = 2.446, 95% CI: 1.363–4.389) as independent imaging predictors of EGFR mutations in GGNs (all P<0.05, **Table 3**). Collinearity analysis revealed no significant collinearity among these predictors. Receiver operating characteristic (ROC) curves determined optimal cutoff values as follows: maximum diameter at 15.25 mm, mean CT attenuation at −412.50 HU, and CTR at 43.50%, based on Youden’s index.

**Table 3:** Multivariable logistic regression of clinical and CT finings and EGFR mutation status of ground glass nodules.

Variables	Univariate		Multivariate	
	OR (95%CI)	*P*	OR (95%CI)	*P*
Gender, n (%)				
male	1.00 (Reference)			
female	1.31 (0.96 ~ 1.80)	0.089		
Smoking history, n (%)				
No	1.00 (Reference)		1.00 (Reference)	
Yes	0.70 (0.51 ~ 0.98)	0.037	0.759 (0.491 ~ 1.172)	0.213
Vacuole sign, n (%)				
No	1.00 (Reference)			
Yes	1.15 (0.75 ~ 1.74)	0.524		
Spiculation, n (%)				
No	1.00 (Reference)			
Yes	1.33 (0.99 ~ 1.80)	0.058		
lobulation, n (%)				
No	1.00 (Reference)		1.00 (Reference)	
Yes	1.40 (1.01 ~ 1.93)	0.042	1.388 (0.910 ~ 2.117)	0.127
Pleural traction sign, n (%)				
No	1.00 (Reference)		1.00 (Reference)	
Yes	1.53 (1.08 ~ 2.16)	0.017	1.351 (0.858 ~ 2.128)	0.194
Vascular convergence sign, n (%)				
No	1.00 (Reference)		1.00 (Reference)	
Yes	1.68 (1.24 ~ 2.29)	<.001	1.632 (1.093 ~ 2.438)	0.017
Air bronchogram sign, n (%)				
No	1.00 (Reference)		1.00 (Reference)	
Yes	2.31 (1.50 ~ 3.57)	<.001	2.446 (1.363 ~ 4.389)	0.003
age(years)	0.99 (0.97 ~ 1.00)	0.087		
mean CT value (HU)	1.01 (1.01 ~ 1.02)	<.001	1.010 (1.008 ~ 1.012)	<.001
maximum diameter (mm)	1.35 (1.28 ~ 1.42)	<.001	1.178 (1.113 ~ 1.247)	<.001
CTR (%)	1.04 (1.03 ~ 1.05)	<.001	1.025 (1.015 ~ 1.035)	<.001

### Construction and validation of nomogram models

**Figure 2:**
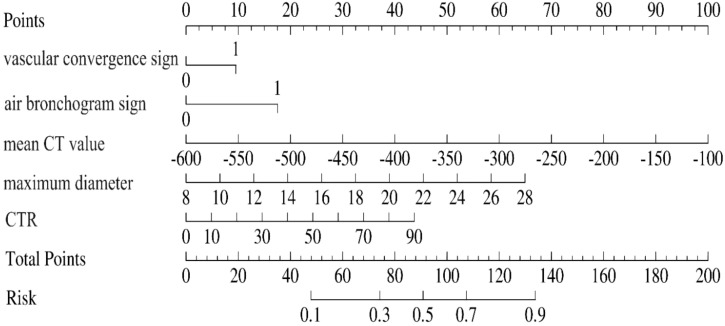
A nomogram model predicting EGFR mutation status in GGN patients.

**Figure 3:**
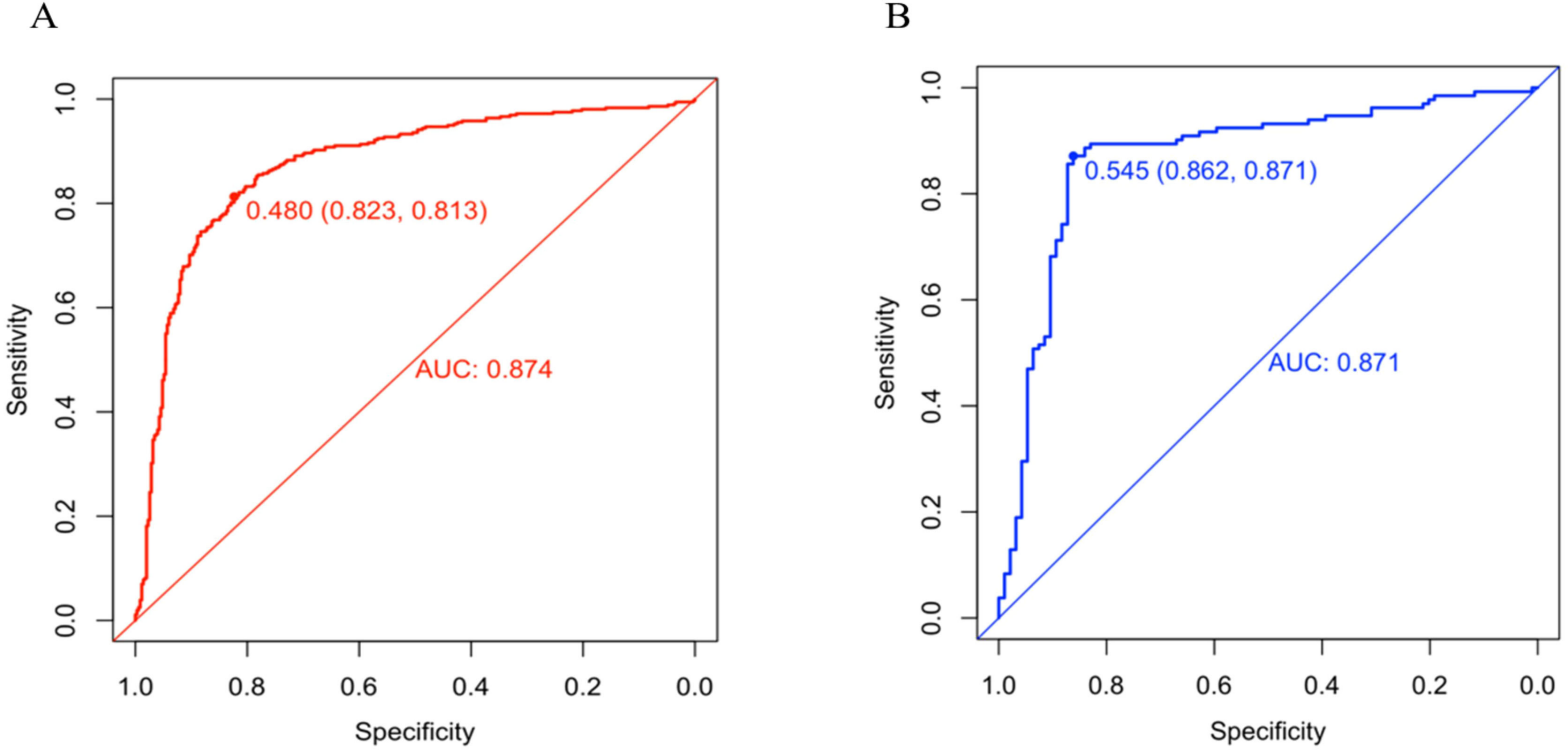
**(A)** ROC curve of the nomogram in training group. **(B)** ROC curve of the nomogram in validation group.

**Figure 4:**
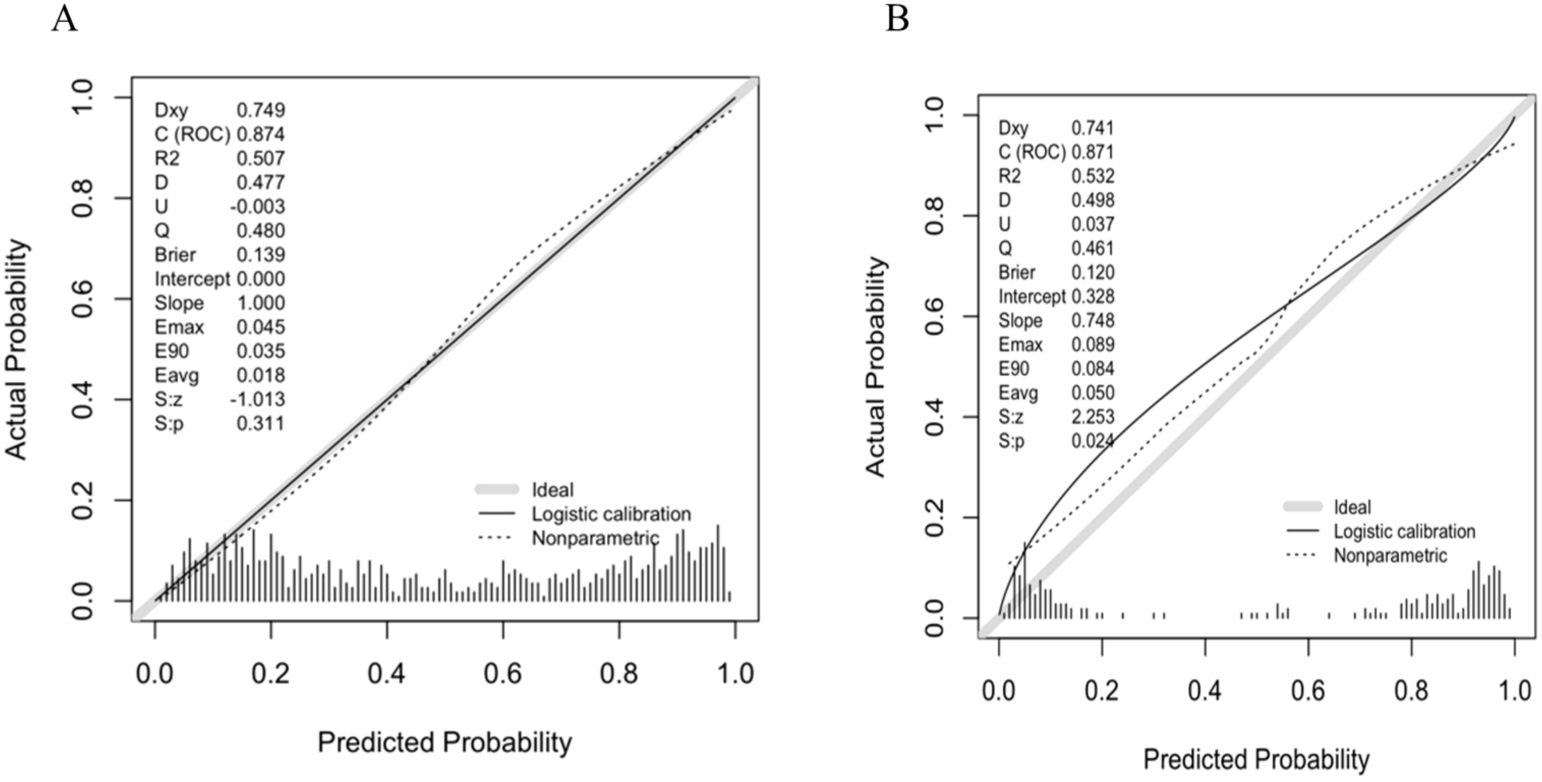
**(A)** Calibration curve of the nomogram in training group. **(B)** Calibration curve of the nomogram in validation group.

**Figure 5:**
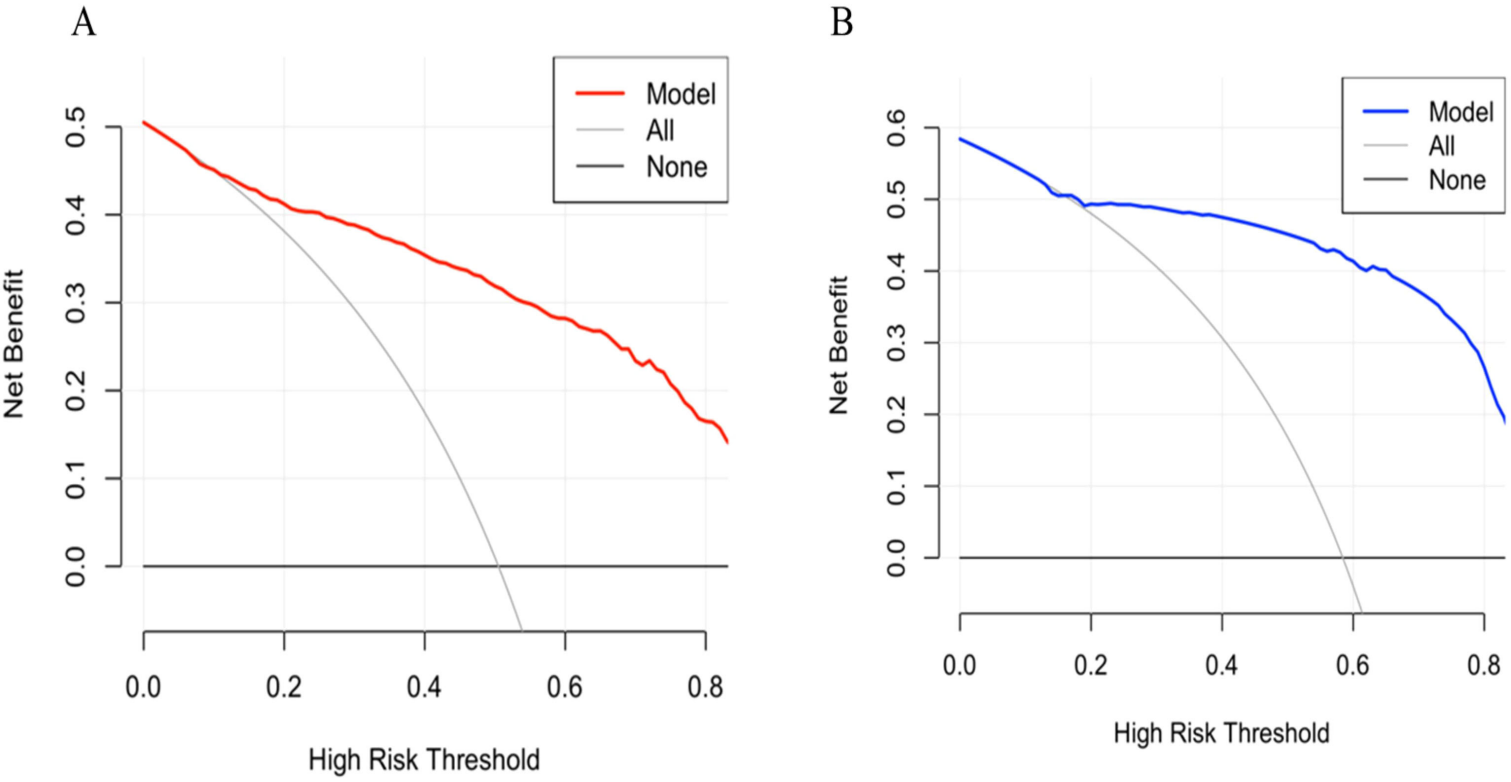
**(A)** Decision curve analysis of the nomogram in training group. **(B)** Decision curve analysis of the nomogram in validation group.

A predictive nomogram incorporating maximum diameter, CTR, mean CT value, air bronchogram sign, and vascular convergence sign was developed (**Figure 2**). The resulting model achieved excellent discrimination, with AUCs of 0.87 (95% CI: 0.85–0.90) in the training group and 0.87 (95% CI: 0.82–0.92) in the validation group (**Figure 3**). Calibration plots confirmed good consistency between predicted probabilities and actual outcomes in both cohorts (**Figure 4**). Decision curve analysis highlighted the nomogram’s substantial clinical utility (**Figure 5**). Internal bootstrap validation (1,000 repetitions) resulted in an AUC of 0.89 (95%CI:0.86–0.92), reaffirming robust model discrimination.

## Discussion

Lung cancer remains the leading cause of cancer incidence and mortality globally, with adenocarcinoma as the most common histological subtype (1, 25). With the widespread use of high-resolution CT, an increasing number of MPLC presenting as GGNs are detected (2). Surgical treatment remains the primary therapeutic option for MPLC (26). For the management of residual lesions, targeted therapy has shown significant potential (6). Previous studies indicated that these ground-glass lesions carry a high frequency of EGFR mutations, and EGFR-TKIs demonstrated favorable therapeutic outcomes for these lesions (7, 8).

With advancements in molecular cancer biology, the development of therapies targeting oncogenic alterations and associated signaling pathways has become a crucial aspect of cancer treatment (27). EGFR mutations are a key oncogenic alteration in lung adenocarcinoma, and inhibitors targeting this mutation have demonstrated promising therapeutic efficacy (28). However, EGFR mutations also contribute to immunotherapy resistance. Previous studies have shown that immune checkpoint inhibitors (ICIs) significantly improve the survival of patients with lung adenocarcinoma without driver gene mutations (29, 30). In contrast, monotherapy with ICIs has not significantly improved efficacy in EGFR-mutant lung adenocarcinoma patients (31). This lack of efficacy may be attributed to factors such as low PD-L1 expression, low tumor mutational burden (TMB), and the upregulation of an immunosuppressive tumor microenvironment (TME), which collectively put EGFR-mutant patients at a disadvantage when receiving ICI treatment (32). Furthermore, studies suggest that EGFR mutations play a pivotal role in the evolution of lung adenocarcinoma, enhancing the tumor’s ability to adapt to existing therapies and leading to diminished therapeutic efficacy and the development of resistance (33, 34). The effects of EGFR mutations have led researchers, in their exploration of cancer treatment strategies, to not only focus on tumor cells but also emphasize the importance of the tumor microenvironment (35). However, our study specifically focuses on refining cell-intrinsic prediction, particularly in determining which patients are likely to benefit from EGFR TKIs. Therefore, accurately predicting the EGFR mutation status is crucial.

However, traditional biopsy techniques have inherent limitations when assessing EGFR mutation status in GGNs, underscoring the need for non-invasive, convenient, and accurate predictive methods (12). Predictive modeling approaches have emerged as promising alternatives to address these challenges (36, 37). Accordingly, this study established a nomogram incorporating multiple radiological parameters to non-invasively predict EGFR mutations, thus facilitating clinical decision-making.

Tumor diameter is an important CT imaging feature of GGNs. Lee et al. demonstrated that tumor diameters were significantly associated with EGFR mutations (38). Similarly, Yang et al. reported a correlation between GGN diameter and EGFR mutation status (39). Our results confirmed that maximum lesion diameter independently predicts EGFR mutation occurrence in GGNs. When the maximum diameter of ground glass nodules exceeds 15.25 mm, the likelihood of EGFR mutation significantly increases. This finding is consistent with the study by Yang et al (39). Lee et al. observed that when the diameter exceeds 2.43 cm (38), the probability of EGFR mutation is highest, which aligns with the results of Usuda et al (40). The differences in cutoff values may be due to variations in sample inclusion criteria. In our study, we specifically included ground-glass nodules, whereas other studies included a broader range of nodule types. This finding aligns with previous reports. Nonetheless, Cheng et al.’s meta-analysis identified no clear correlation between lesion size and EGFR mutation status, potentially attributable to confounding variables affecting tumor dimensions within the reviewed studies (41).

As the proportion of the solid component increases, GGNs exhibit progressively greater invasiveness and more prominent malignant characteristics (42). Previous studies have shown that the presence of a ground-glass component correlates with a higher rate of EGFR mutations in non-small cell lung cancer (NSCLC) (43, 44). However, limited research has specifically addressed the relationship between changes in the relative proportions of ground-glass and solid components and EGFR mutation status in GGNs. CTR is currently recognized as a critical imaging feature for evaluating GGNs (45). In our study, we observed that a higher proportion of solid components increased the likelihood of EGFR mutations, consistent with previous reports. The optimal cutoff value of CTR for diagnosing EGFR mutations was identified as 43.50%,which is consistent with previous studies (46).

With increasing tumor invasiveness, tumor cells progressively infiltrate surrounding normal tissue structures, resulting in an increased mean CT value of GGNs. Mean CT value plays an important role in evaluating GGNs (47, 48). However, whether the mean CT value can predict EGFR mutation status remains unclear. Our findings first indicate that the mean CT value is an independent predictor of EGFR mutation status in GGNs. Specifically, when the mean CT value exceeds -412.50 HU, the likelihood of EGFR mutation significantly increases, achieving strong diagnostic performance. Zhan et al. pointed out that (48)the likelihood of invasive adenocarcinoma increases when the mean CT value of ground-glass nodules exceeds -449.5 Hu, a view that is also supported by other researchers (49). This is consistent with the cutoff value for EGFR mutations in GGNs observed in our study, suggesting that as the degree of invasion in GGNs increases, the likelihood of EGFR mutations also rises, aligning with the perspectives of previous studies (50).

Qualitative CT imaging characteristics are also valuable for predicting EGFR mutations. Liu et al. identified the vascular convergence sign as indicative of EGFR mutations (51). Likewise, Cao et al. demonstrated that the presence of this sign elevates the mutation risk by approximately 2.26-fold (52). A subsequent meta-analysis by Zhang et al. further supported this result (44). Our findings also confirmed the independent predictive value of the vascular convergence sign for determining EGFR mutation status. However, Zou et al. reported contradictory results, possibly due to their small sample size (13). Rizzo et al. found that the air bronchogram sign could predict EGFR mutation status (15). A similar finding was reported by Sabri et al. and subsequently confirmed by Zhang et al., who included 2,380 patients in their study. Our research yielded similar results, indicating that the air bronchogram sign significantly increases the likelihood of EGFR mutation.

Previous studies demonstrated that gender, smoking history, and CEA levels play important roles in predicting EGFR mutation status (44, 53). However, in our study, none of these factors—gender, smoking history, or CEA levels—showed predictive value. This discrepancy may arise because prior studies focused primarily on non-small cell lung cancer, whereas our research specifically targeted lung adenocarcinoma presenting as GGNs. This subtype occurs predominantly in younger, non-smoking women, and their CEA levels are typically normal. Therefore, significant differences in gender, smoking history, and CEA levels between groups may not have been evident in our cohort.

Compared with traditional approaches relying on single predictors, a nomogram model integrating multiple features improves prediction accuracy and efficiency (54, 55). Recently, some researchers have developed radiomics-based models to predict EGFR mutation status in GGNs (56). Although radiomics is increasingly used in clinical research, differences in resource availability and infrastructure may still affect its implementation across various regions. Alternatively, other studies utilized more accessible clinical and imaging data to assess EGFR mutation status in GGNs (36). However, these previous studies often involved small sample sizes, and their predictive models lacked sufficient validation, underscoring the need for further investigation. The present investigation provides a comprehensive assessment of clinical and radiological predictors associated with EGFR mutation status using a substantial sample size of GGN cases. Notably, we identified mean CT value as an independent predictor for EGFR mutations, an observation not previously reported. Additionally, we successfully developed and validated a predictive nomogram model by combining multiple features. Upon patient admission, clinicians can assess the five independent predictive factors (maximum diameter, CTR, mean CT value, air bronchogram sign, and vascular convergence sign). A total score is then calculated based on the individual scores of these factors to estimate the EGFR mutation status of ground-glass nodules. If a ground-glass nodule is determined to harbor an EGFR mutation, EGFR-targeted therapy may be considered for the management of the remaining lesions in patients with multiple GGNs, thus supporting the precision treatment of lung adenocarcinoma.

Our predictive model demonstrated good performance. However, with advancements in technology, multimodal integrative analysis combining imaging models with genomic or transcriptomic data has become an emerging trend (57, 58). Du et al. developed a prognostic model by integrating imaging and transcriptomic data, achieving an AUC of 0.9 (59). Liu et al. constructed a predictive model by combining molecular and clinical features, which also improved predictive performance (60). Wang et al. built a prognostic model for hepatocellular carcinoma by integrating transcriptomic data with CT features, achieving an AUC of 0.834 (61). In our future research, we plan to perform transcriptome sequencing to identify key genes associated with EGFR mutations in ground-glass nodules, and to integrate these with imaging features to develop a multimodal diagnostic model for EGFR mutation.

Recent advances in clinical cancer genomics have highlighted the need to systematically annotate and prioritize somatic variants with established therapeutic relevance. A recent ClinGen somatic curation effort has initiated the annotation of EGFR variants, aiming to define their clinical actionability (62). A recent case report has identified EML4-ALK variant 3a/b as a mechanism of acquired resistance to osimertinib in a patient with EGFR L858R-positive non-small cell lung cancer (63). These findings underscore that accurate prediction of mutation status has direct implications for therapeutic decision-making and resistance monitoring. Our model, by non-invasively predicting EGFR mutation status in GGN lesions, may help identify patients likely to benefit from targeted therapy, thereby enhancing its translational potential. Future integration of curated variant annotations could further expand its utility in anticipating resistance pathways and guiding individualized treatment strategies.

Recent studies have highlighted cancer cell plasticity as a critical contributor to therapeutic resistance (64). Plasticity refers to the ability of tumor cells to adopt alternative phenotypes in response to selective pressures, including drug treatment (65). This dynamic adaptability, often driven by intrinsic signaling pathways such as Notch, Wnt, MAPK, PI3K, and STAT3, allows subpopulations of cancer cells to evade therapy by transitioning into drug-tolerant or stem-like states (66). Such phenotypic switching underlies intratumoral heterogeneity and has been identified as a key mechanism of acquired resistance to targeted therapies. While our model provides a non-invasive method to predict EGFR mutation status at baseline, it is important to recognize that mutation status alone may not capture the full spectrum of tumor adaptability. Integrating temporal imaging data or molecular follow-up in future modeling efforts may enhance the ability to anticipate resistance development and better support individualized therapeutic strategies.

Despite promising outcomes, this study has certain limitations. First, The retrospective design may inherently introduce selection biases, and data collection from a single center could limit the generalizability of results. Although internal validation using temporally distinct cohorts was conducted, multicenter and prospective studies are warranted to further validate and generalize the findings. Second, imaging measurements in this analysis involved manual assessments, inevitably resulting in potential measurement variability. Finally, Our study focused exclusively on lung adenocarcinomas presenting as ground-glass nodules, a highly selected subgroup with relatively indolent behavior and distinct molecular features. As a result, the generalizability of our model to broader or more heterogeneous NSCLC populations remains uncertain and warrants future investigation.

## Conclusion

In conclusion, the constructed nomogram integrating maximum diameter, CTR, mean CT value, air bronchogram sign, and vascular convergence sign effectively predicts EGFR mutation status in GGNs. The proposed model exhibits robust predictive capability and holds potential for guiding personalized clinical decisions and patient management in clinical practice.

## Data Availability

The raw data supporting the conclusions of this article will be made available by the authors, without undue reservation.
